# Supplementary material for: Flavonoids from Mulberry Leaves Alleviate Lipid Dysmetabolism in High Fat Diet-Fed Mice: Involvement of Gut Microbiota
Source: Microorganisms. 2020 Jun 7;8(6):860. doi: 10.3390/microorganisms8060860 (PMC7355566; doi:10.3390/microorganisms8060860)
Supplement: Supplementary file 1 [file microorganisms-08-00860-s001.pdf]

Supplementary Figure 1 The overall protocol used in this study.

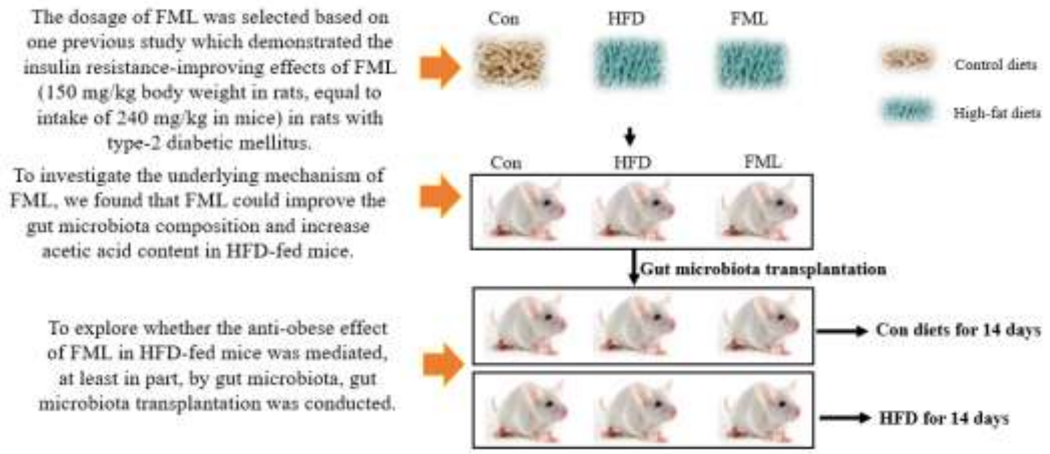

Supplementary Figure 2 Schematic diagram of microbial transplantation.

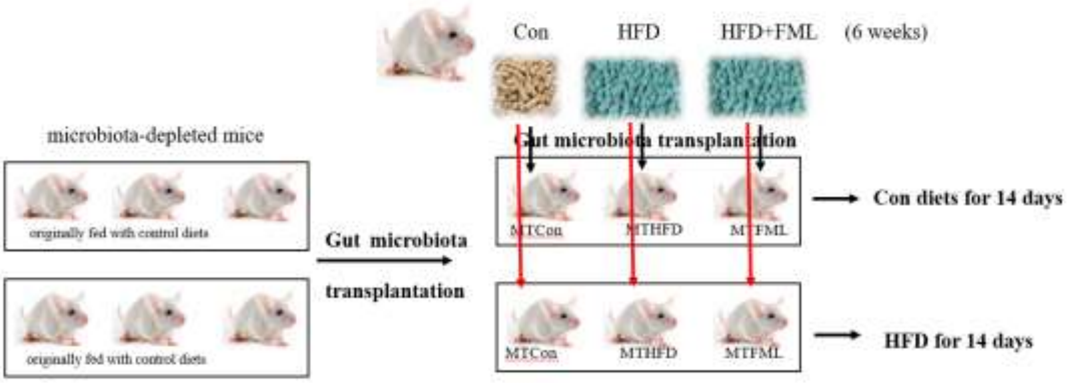

Supplementary Table 1 Primers used in this study.

Gene	Nucleotide sequence (5-3′)
SREBP1	F:GAACGACATCGAAGACATGC<br>R:GAGAAGCTCTCAGGAGAG
SREBP2	F: GTGCGCTCTCGTTTTACTGAAGT<br>R:GTATAGAAGACGGCCTTCACCAA
PPARα	F： AGGCTGTAAGGGCTTCTTTCG<br>R： GGCATTTGTTCCGGTTCTTC
PPARγ	F:CCATTCTGGCCCACCAAC<br>R:AATGCGAGTGGTCTTCCATCA
ACC	F:TTCAGTTCATGCTGCCCACA<br>R:AGGTTGGAGGCAAAGGACAT
LXRα	F:CTCAATGCCTGATGTTTCTCCT<br>R:TCCAACCCTATCCCTAAAGCAA
LXRβ	F:GATCCTCCTCCAGGCTCTGAA<br>R:TGCGCTCAGGCTCATCCT
β-actin	F:GTCCACCTTCCAGCAGATGT<br>R:GAAAGGGTGTAAAACGCAGC

Supplementary Table 2

The quantification values of Glucose, TG, CHOL, HDL and LDL.

Item	Con	HFD	FML	SEM	p-value
Glucose, mmol/L	2.39^{b}	4.04^{a}	4.36^{a}	0.33	<0.01
TG, mmol/L	1.93^{b}	2.23^{a}	1.85^{b}	0.18	0.02
CHOL, mmol/L	3.31^{b}	4.69^{a}	4.07^{ab}	0.30	<0.01
HDL, mmol/L	2.68^{c}	4.45^{a}	3.56^{ab}	0.30	<0.01
LDL, mmol/L	0.35	0.41	0.36	0.10	0.36

^{a,b,c}Within a row, values with different superscripts differ significantly at $P < 0.05$ and a trend toward significance at $P < 0.10$. Data are expressed as means ± SEM, n=7.

Supplementary Table 3 Effects of FML on total reads and clean reads of microbiota.

#Sample_name	Raw_reads(#)	Clean_Reads(#)	Base(nt)	AvgLen(nt)	Q20	GC%	Effective%
Con1	85116	80095	32628985	407	80.7	53.44	94.1
Con2	88355	80216	32887493	409	79.97	52.3	90.79
Con3	79495	75923	31103293	409	78.71	52.31	95.51
Con4	87134	80161	32544602	405	80.17	52.7	92
Con5	82504	80187	32748416	408	80.25	53.12	97.19
Con6	86698	80208	32682130	407	79.26	51.89	92.51
Con7	88287	80154	32987932	411	79.57	53.07	90.79
Con8	52286	50426	20587651	408	79.02	50.46	96.44
HFD1	84206	80022	32784881	409	80.64	52.66	95.03
HFD2	53401	50577	20698809	409	80.18	53.11	94.71
HFD3	83091	80112	32265000	402	79.11	53.61	96.41
HFD4	84467	80139	33137223	413	81.1	51.22	94.88
HFD5	54029	51833	21668115	418	84.68	49.82	95.94
HFD6	82175	80053	33563240	419	82.62	49.99	97.42
HFD7	69809	65040	26763103	411	83	52.96	93.17
HFD8	85902	80090	33096967	413	83.34	53.24	93.23
FML1	53045	51569	21652221	419	83.91	50.26	97.22
FML2	82240	80219	33479790	417	84.28	51.02	97.54
FML3	88458	80096	33296765	415	81.93	51.09	90.55
FML4	87368	80040	33197615	414	81.25	51.79	91.61
FML5	87072	80122	33281835	415	81.13	51.58	92.02
FML6	85995	80303	33010360	411	83.3	53.32	93.38
FML7	87180	80070	33181692	414	83.6	52.6	91.84
FML8	82446	80068	33083081	413	83.81	52.95	97.12

Supplementary Table 4

The quantification values of Glucose, TG, CHOL, HDL and LDL.

**Item**	**Con**	**HFD**	**FML**	**SEM**	***P*-value**
Glucose, mmol/L	6.1^{b}	7.05^{ab}	7.52^{a}	0.35	0.02
TG, mmol/L	1.63^{a}	1.26^{b}	1.57^{ab}	0.21	0.09
CHOL, mmol/L	3.20^{b}	4.59^{a}	4.63^{a}	0.34	<0.01
HDL, mmol/L	2.66^{b}	3.97^{a}	4.07^{a}	0.33	<0.01
LDL, mmol/L	0.48	0.54	0.50	0.12	0.59

^{a,b} Within a row, values with different superscripts differ significantly at $P < 0.05$ and a trend toward significance at $P < 0.10$. Data are expressed as means ± SEM, n=7.

Supplementary Table 5

The quantification values of Glucose, TG, CHOL, HDL and LDL.

Item	Con	HFD	FML	SEM	*P*-value
Glucose, mmol/L	6.30	6.80	6.33	0.37	0.54
TG, mmol/L	0.98	1.11	1.02	0.16	0.39
CHOL, mmol/L	3.55	4.09	3.41	0.32	0.2
HDL, mmol/L	3.18	3.61	3.15	0.32	0.42
LDL, mmol/L	0.32[b]	0.51[a]	0.37[ab]	0.15	0.07

[a,b] Within a row, values with different superscripts differ significantly at $P < 0.05$ and a trend toward significance at $P < 0.10$. Data are expressed as means ± SEM, n=7.

Supplementary Table 6 The quantification values for SCFA.

Item	Con	HFD	FML	SEM	*P*-value
**Acetic acid, μg/g**	2219.85[a]	1776.49[b]	2100.22[ab]	6.24	0.04
**Propionate, μg/g**	445.40[a]	339.54[b]	372.81[ab]	3.00	0.03
**Isobutyric acid, μg/g**	32.58	39.96	34.36	0.98	0.20
**Butyrate, μg/g**	283.12[a]	251.63[ab]	220.60[b]	2.25	0.03
**Isovaleric acid, μg/g**	34.044[b]	53.78[a]	44.601[ab]	1.33	0.05
**Valerate, μg/g**	43.72[b]	66.95[a]	43.62[b]	1.43	0.02

[a,b] Within a row, values with different superscripts differ significantly at $P < 0.05$,Data are presented as mean ± SEM, n=9.
